# Supplementary material for: Content-rich biological network constructed by mining PubMed abstracts
Source: BMC Bioinformatics. 2004 Oct 8;5:147. doi: 10.1186/1471-2105-5-147 (PMC528731; doi:10.1186/1471-2105-5-147)
Supplement: Additional File 2 — The original results of the above study (non-essential files are deleted to keep the file size under the limit set by BMC bioinformatics). [file 1471-2105-5-147-S2.bz2 › chilibotAdditionalFile2/dip20/14ID9153399E59/html/ITGB2_ICAM2.html]

 


 **ITGB2** and **ICAM2** 
  
Found 158 abstracts in PubMed, retrieved 20.  
 

 What does Google say? 
 PDF only 
| .edu only 

---

**Interactive relationship** (e.g. stimulation, inhibition, etc)

**Neutral relationship**- We show that this process is dependent on an initial interaction between alphaLbeta2 integrin CD11a CD18  [ **ITGB2** ]  on infected monocytic cells and intercellular adhesion molecule 2  [ **ICAM2** ]  ICAM 2  [ **ICAM2** ] .  Ref: 11739669 J Virol, 2002
- The beta 2 integrin LFA 1  [ **ITGB2** ]  CD11a CD18  [ **ITGB2** ]  is a leukocyte specific adhesion molecule that mediates leukocyte extravasation, antigen presentation, and T cell mediated cytolysis through its interaction with its counter receptors, ICAM 1, ICAM 2  [ **ICAM2** ] , and ICAM 3.  Ref: 12799043 J Immunol Methods, 2003

**Non-interactive relationship** (e.g. studied together, co-existance, homology, etc.)

- Pretreatment of human PMN with anti CD18  [ **ITGB2** ]  mAb or pretreatment of HUVEC with a combination of three mAbs against ICAM 1, ICAM 2  [ **ICAM2** ] , and E selectin inhibited by 85% the MC dependent PMN transmigration.  Ref: 12370389 J Immunol, 2002
- ICAM 2  [ **ICAM2** ]  engagement by CD19 cells of its natural receptor, LFA 1  [ **ITGB2** ] , on CD4 naive cells specifically induced AKT activity in the absence of an MHC peptide interaction.  Ref: 11825565 Immunity, 2002
- Agglutination induced by A80 was inhibited by stromal cell derived factor 1, T22, and phorbol 12 myristate 13 acetate but was not significantly altered by pretreatment of cells with pertussis toxin, wortmannin, or MAbs to LFA 1  [ **ITGB2** ] , ICAM 1, ICAM 2  [ **ICAM2** ] , and ICAM 3.  Ref: 11689635 J Virol, 2001
- All the cytokines tested interleukin 1beta IL 1beta, IL 2, IL 4, IL 7, IL 10, interferon gamma IFN gamma as well as CAMs ICAM 1, ICAM 2  [ **ICAM2** ] , VCAM 1, LFA 1  [ **ITGB2** ]  and CD44 were expressed at substantial levels in both spleen and LNs.  Ref: 12193230 Scand J Immunol, 2002
- Blocking monoclonal antibodies were used against the platelet and neutrophil surface receptors glycoprotein complex IIb IIla, P selectin, ICAM 2  [ **ICAM2** ] , CD11a, CD11b, and CD18  [ **ITGB2** ] .  Ref: 12069189 Shock, 2002
- They expressed CD4, CD11a, CD11b, CD11c, CD18  [ **ITGB2** ] , CD32, CD33, CD45R0, CD48, CD50, CD54, CD58, CD64, CD86, CD102  [ **ICAM2** ] , CD116, CD123, and HLA DR.  Ref: 11698124 Exp Hematol, 2001
- Linear and cyclic peptides derived from the sequences of LFA 1  [ **ITGB2** ] , ICAM 1, ICAM 2  [ **ICAM2** ] , VCAM 1, and FN C1 have been shown to have inhibitory effects in vitro and in vivo.  Ref: 11857637 Med Res Rev, 2002
- Although the integrin LFA 1  [ **ITGB2** ]  is a counter receptor for both ICAM 2  [ **ICAM2** ]  and ICAM 3, on DC, DC SIGN is the high affinity adhesion receptor for ICAM 2  [ **ICAM2** ]  3.  Ref: 12234361 Immunol Rev, 2002
- ICAM 1 antibody prevented the extravasation, while antibodies to other adhesion molecules showed little CD18  [ **ITGB2** ] , ICAM 2  [ **ICAM2** ]  or no inhibition CD11a and ICAM 3.  Ref: 11442494 Br J Haematol, 2001
- IL 1beta is a co stimulator in activating Th cells, which manifests its activity together with LFA 1  [ **ITGB2** ] , ICAM 1 and ICAM 2  [ **ICAM2** ] .  Ref: 12400871 Int Immunopharmacol, 2002
- RESULTS Specific cation dependent binding of lymphocytes to bronchial endothelium was observed which was significantly inhibited by antibodies against P selectin, PSGL 1, L selectin, LFA 1  [ **ITGB2** ] , ICAM 1 and ICAM 2  [ **ICAM2** ]  but not E selectin, VLA 4, VCAM 1 or Mac 1.  Ref: 12454301 Thorax, 2002
